# Supplementary material for: Evaluation of SARS-CoV-2 antibody point of care devices in the laboratory and clinical setting
Source: PLoS One. 2022 Mar 31;17(3):e0266086. doi: 10.1371/journal.pone.0266086 (PMC8970483; doi:10.1371/journal.pone.0266086)
Supplement: S1 Table — (DOCX) [file pone.0266086.s001.docx]

| **Company** | **Product specifications** | **Manufacturer's Sensitivity** | **Manufacturer's Specificity** |
| --- | --- | --- | --- |
| **Abingdon Health Laboratories**  2007003 Antibody type: IgG  Target antigen: Spike (S) protein | Transfer 2.5µL of venous whole blood or serum or 5µL capillary blood to sample well. Add 100µL buffer solution. Read results at 20 minutes. | 98.03% (95% CI: 95.03%-99.46%)  N= 203 positive cases on commercial IgG SARS-COV-2 ELISA kit | 99.56% (95% CI: 98.4%-99.95%)  N= 450 pre-pandemic samples |
| **Alpha Pharma**  VID35 08 012 LOT E2004009 Antibody type: IgG and IgM Target antigen: S protein | Transfer 10µL sample (venous or capillary whole blood or serum) to sample well. Add 2 drops (60-80µL) buffer. Read result at 15 minutes- don't read after 20 minutes. | Infection time 4-10 days: 81.25% Infection time 11-24 days: 97.1 % N=397 PCR positive cases | 100% N= 128 PCR negative samples |
| **Biomerica**  kit 1507A-50, BATCH 6630 Antibody type: IgG and IgM Target antigen: Nucleocaspid (N) protein | Transfer 10µL serum or 20µL venous or capillary whole blood to sample well. Add 2 drops (80µL) buffer. Read at 10 minutes. Do not interpret results after 20 minutes. | 85.0% (95%CI: 62.1%-96.8%) N= 20 PCR positive cases | 96.3% (95%CI: 89.4%-99.2%) N= 80 PCR negative cases |
| **Biozek** BNCP402E00084 Antibody type: IgG/IgM Target antigen: not disclosed | Transfer 20µL whole blood or 10µL serum/plasma to sample well. Apply 2 drops (80µL) buffer. Read results at 10 minutes. Do not interpret after 20 minutes. | IgG 100% (95% CI: 86.0-100%)  IgM 85.0% (95%CI: 62.1-96.8%) N=20 PCR positive cases | IgG 98.0% (95%CI: 89.4%-99.9%) IgM 96.0% (95%CI: 86.3-99.5%) N=50 negative PCR cases |
| **Fortress** COV19-OTC-2004168-1 Antibody type: IgG and IgM Target antigen: S protein | Transfer 5-10µL whole blood, serum or plasma into the sample well. Add 2 drops of buffer and read results at 10 minutes. | 95.6% (90.7- 98.4%) N=137 | 95.2% (91.5-97.7%) N= 209 |
| **Jiangsu** 28d2001 Antibody type: IgG/IgM Target Antigen: S protein | Transfer 20µL blood into sample diluent. Shake well then add 5 drops (100µL) of diluent/sample mixture to sample well. Read result at 3 minutes. | 97.14% (95% CI: 90.17%~99.21%) N=70 positive samples | 100% (95% CI: 93.98%~100.00%)  N=60 negative samples |
| **Lepu** 20CG2508x Antibody type: IgG/IgM Target Antigen: not disclosed | Transfer 10µL serum or plasma or 20µL whole blood to sample well. Add 2 drops (80µL) buffer. Read test result at 10-20 minutes. Result should not be interpreted after 20 minutes. | IgG sensitivity: 100%  N=92 Positive samples with reference LFIA method IgM sensitivity: 97%  N= 72 positive samples reference LFIA method | IgG specificity: 99%  N=128 negative samples with reference LFIA method IgM specificity 100% N=147 negative samples with reference LFIA method |
| **Menarini / Healgen** GCCOV402A (2005156) Antibody type: IgG/IgM Target antigen: S1, S2 and N protein | Transfer 5µL serum/plasma or 10µL whole blood to sample well. Add 2 drops (80µL) buffer to buffer well. Read at 10 minutes and no later than 15 minutes. | IgM sensitivity 87.9% (87/99)  N= 99 PCR positive samples IgG sensitivity 97.2% (35/36) during the convalescence period | IgG/IgM specificity is 100%( 14/14)  N=14 PCR negative samples |
| **Menarini 2** GCCOV402A (2003288) Antibody type: IgG/IgM Antigen target: S1, S2 and N protein | Transfer 5µL serum/plasma or 10µL whole blood to sample well. Add 2 drops (80µL) buffer to buffer well. Read at 10 minutes and no later than 15 minutes. | IgM sensitivity 87.9% (87/99)  N= 99 PCR positive samples IgG sensitivity 97.2% (35/36) during the convalescence period | IgG/IgM specificity is 100%( 14/14)  N=14 PCR negative samples |
| **Mologic/ Visitec** 7066351 Antibody type: IgG/IgM/IgA Antigen target: S2, RBD (receptor binding domain), N protein | Transfer 5µL whole blood/plasma/serum to sample well. Add 2 drops of buffer. Read results at 10 minutes. | 96% (79.65-99.9%) N=25 PCR confirmed cases | 98.8% (99.6-99.8%) N=257 pre-COVID 19 pandemic samples |
| **Roche** 9901-ncov-02c qc07920001 Antibody type: IgG/IgM Target Antigen: S protein, N protein | Transfer 20µL whole blood or 10µL serum/plasma to sample well. Apply 3 drops (90µL) buffer. Read results between 10 and 15 minutes. | 7-14 days 92.59% (82.11-97.94%)  N= 54 PCR positive samples >14 day sensitivity 99.03% (94.71-99.98%) N=103 PCR positive samples | 98.65% (96.1-99.72%) N=222 PCR negative samples |
| **Pharmact** 1797 6618 Anti-body type: IgG/IgM Target antigen: not disclosed | Transfer 50µL whole blood or serum to sample well. Add 2 drops of buffer. Read at 20 minutes. | 4-10 days symptom onset: 70% for IgM 11-24 days symptom onset: 92.3% for IgM 11-24 days symptom onset: 98.6% for IgG | 100% N=126 |
| **idsolid**  No batch numbers on kits Antibody type: IgG/IgM Antigen target: not disclosed | Transfer 20µL whole blood or 10µL serum/plamsa to sample well. Apply 2 to 3 drops of buffer (about 100µL). Read results at 15 minutes. | IgM sensitivity 96% (48/50) IgG sensitivity 98.2% (56/57) reference test PCR | IgM specificity 100% (100/100) IgG specificity 100% (100/100) |
| **Wuhan Life Origin Biotech/Szybio** C200525001/SF20025 Antibody type: IgG/IgM Target Antigen: not disclosed | Transfer 20µL whole blood or 10µL serum/plamsa to sample well. Apply 2 drops (60µL) buffer. Read result at 15 minutes and no later than 18 minutes. | not reported | not reported |
| **Wuhan Easy diagnostics** SA-2-D 20050602 Antibody type: IgG/IgM Target Antigen: not disclosed | Transfer 10µL plamsa/serum or 15µL whoel blood to sample well. Add 2 drops (70µL) sample diluent. Read result at 10 minutes. | 100% (95% CI: 100-100%) N=207 PCR positive samples | 99.56% (95% CI: 98.94-100%) N=229 PCR negative samples |
